# Perspectives of health professionals on physical activity and sedentary behaviour in hospitalised adults: A systematic review and thematic synthesis

**DOI:** 10.1177/02692155231170451

**Published:** 2023-04-17

**Authors:** Tahlia Alsop, James Woodforde, Ingrid Rosbergen, Niruthikha Mahendran, Sandra Brauer, Sjaan Gomersall

**Affiliations:** 1 School of Health and Rehabilitation Sciences, 1974 The University of Queensland, Brisbane, Australia; 2 School of Human Movement and Nutrition Sciences, 1974 The University of Queensland, Brisbane, Australia; 3 Department of Physiotherapy & Faculty of Health, 125778 University of Applied Sciences Leiden, Leiden, The Netherlands

**Keywords:** Physical activity, sedentary behaviour, hospitalised adults, inpatient setting, health professionals

## Abstract

**Objective:**

To explore health professionals’ perspectives on physical activity and sedentary behaviour of hospitalised adults to understand factors that contribute to these behaviours in this environment.

**Data sources:**

Five databases (PubMed, MEDLINE, Embase, PsycINFO and CINAHL) were searched in March 2023.

**Review methods:**

Thematic synthesis. Included studies explored perspectives of health professionals on the physical activity and/or sedentary behaviour of hospitalised adults using qualitative methods. Study eligibility was assessed independently by two reviewers and results thematically analysed. Quality was assessed using the McMaster Critical Review Form and confidence in findings assessed using GRADE-CERQual.

**Results:**

Findings from 40 studies explored perspectives of over 1408 health professionals from 12 health disciplines. The central theme identified was that physical activity is not a priority in this setting due to the complex interplay of multilevel influences present in the interdisciplinary inpatient landscape. Subthemes, the hospital is a place for rest, there are not enough resources to make movement a priority, everyone's job is no one's job and policy and leadership drives priorities, supported the central theme. Quality of included studies was variable; critical appraisal scores ranged from 36% to 95% on a modified scoring system. Confidence in findings was moderate to high.

**Conclusion:**

Physical activity in the inpatient setting is not a priority, even in rehabilitation units where optimising function is the key. A shift in focus towards functional recovery and returning home may promote a positive movement culture that is supported by appropriate resources, leadership, policy, and the interdisciplinary team.

## Introduction

In certain circumstances, hospitalisation is required to deliver effective healthcare. However, even in circumstances of medical necessity, hospitalisation poses a risk of deleterious secondary effects such as functional decline^1–3^ that can persist after discharge.^4^ Even in those who ambulate independently, high sedentary time and low levels of physical activity during hospitalisation is common,^5–7^ with an estimated 87–100% of time spent sitting or lying in bed^8^ and a daily average of only 880 steps.^9^ These patterns are similar even in inpatient settings where the purpose of admission is to optimise recovery, such as rehabilitation. Adults and older adults in inpatient rehabilitation are typically inactive^10–20^ and have high levels of sedentary time^14–16,18,21–23^ which are associated with adverse outcomes including functional decline and hospital-acquired disability.^24–27^ Inpatient environments tend to foster a focus on illness rather than recovery, contrary to modern perspectives that support the optimisation of functional recovery across the care continuum.^28^ Given that greater physical activity is associated with a plethora of improved health and wellbeing outcomes^29^ and supports recovery, the high levels of inactivity are important to investigate in inpatient populations.

To date there has been considerable investigation into the complex and multifactorial determinants of physical activity and sedentary time in the inpatient setting. Previous research exploring patient perspectives found that hospital staff have an important role in facilitating physical activity.^30^ However, patients also reported that staff and the hospital environment can discourage physical activity and promote bed rest.^31^ Evidence that physical activity levels can double immediately post discharge further support that hospital-related factors may limit mobility more than physical capacity.^32,33^ The World Health Organisation (WHO) has established a global action plan to optimise physical activity and sedentary time in hospital settings, citing a need for a ‘whole-of-system’ approach to target these behaviours.^34^

Previous related systematic reviews have found that although health professionals recognise the value of improving these behaviours, there are complex multilevel influences that challenge effective implementation and promotion.^35,36^ Hospital culture, the physical environment, resource constraints and a lack of clarity around roles and responsibilities of team members have been highlighted to play a role.^35,36^ However, previous reviews were not specific to the inpatient setting,^37^ combined patient, clinician and carer perspectives,^35,36^ explored single profession perspectives only^38^ and excluded subacute and rehabilitation settings.^36^ Additionally, previous reviews have captured the perspectives of a relatively limited number of health professionals (*n* = 269^36^ and *n* = 145^35^), possibly due to the diverse language used across disciplines to define physical activity and sedentary behaviour in this context. This review therefore focussed on synthesising health professional perspectives specifically utilising a broad search strategy to account for this diverse language, given the established important role of health professionals in supporting physical activity and sedentary behaviour change in inpatient settings. Therefore, the aim of this systematic review is to explore perspectives of health professionals on physical activity and sedentary behaviour of hospitalised adults (including older adults), using an extensive search strategy, to understand factors that may contribute to these behaviours in patients in the inpatient environment.

## Materials and methods

This systematic review and thematic synthesis has been conducted in accordance with Preferred Reporting Items for Systematic Reviews and Meta-Analyses (PRISMA) guidelines (see Supplemental File 1 for checklist),^39^ and was registered with the International Prospective Register of Systematic Reviews (PROSPERO) on 12/06/2021 (CRD42021254583).^40^

Qualitative studies exploring perspectives of health professionals on physical activity and sedentary behaviour of hospitalised adults were eligible for inclusion. This included mixed-methods studies, however, only the qualitative data were extracted and synthesised. Similarly, where studies also explored other stakeholder perspectives (e.g. patient and unpaid caregivers), only data from health professionals were included. Studies that focused specifically on exercise prescription or structured exercise programmes were excluded, as the purpose of our review was to explore physical activity and sedentary behaviour across the entire 24-hour day, not only structured therapy. Studies based in outpatient, community, or other non-hospital settings, or where health professionals primarily work with paediatric or adolescent populations, were excluded. Hospital settings with special tailored environments such as mental health, oncology, palliative, COVID-19 or intensive care settings were also excluded, given these settings can have context-specific features that may influence physical activity and sedentary behaviour. Where studies included data from multiple settings, populations or wards, data from those areas/populations stipulated in our exclusion criteria were not extracted or used in the analysis.

The following databases were searched from their inception to the 2^nd^ March 2023: PubMed, MEDLINE, Embase, PsycINFO and CINAHL. Searches were restricted to original studies published in English, in peer reviewed journals, with no date restrictions. Search terms included keywords, their synonyms and (where relevant) MeSH terms of ‘physical activity’, ‘sedentary behaviour’, ‘healthcare providers’, ‘hospitals’ and ‘qualitative research’. Synonyms applied were expansive to account for the diverse language used to define physical activity and/or sedentary behaviour across the literature in this population and setting. The full search terms are included in Supplemental File 2.

Following database searches, titles and abstracts of retrieved articles were independently screened by two reviewers (TA, JW) to establish eligibility. Remaining studies were reviewed in full-text independently by two reviewers (TA, SG, NM, IR). A third reviewer from the research team reviewed conflicts as required. Backward citation searching was used to screen reference lists of those studies included after the full-text screening stage. Data were extracted by one reviewer (TA) and checked for accuracy by a second reviewer (NM, IR). Disagreements were resolved by discussion with a third reviewer. Corresponding authors were contacted for further data as required.

Descriptive themes were derived from qualitative data using inductive thematic analysis based on the six stages proposed by Braun and Clarke.^41–43^ Two reviewers (TA, SB) independently became familiar with the data: reading and re-reading extracted data and generating initial ideas. Initial ideas were further refined and coded into preliminary themes based on recurring concepts, then grouped to establish key themes. Then, themes were reviewed across the entire dataset. Themes were further defined and named to produce collated results. All themes identified were discussed by the two reviewers at each of the stages to check for agreement and reach a consensus. This was repeated until the central themes were identified. Once this was completed, a third reviewer (IR) cross-checked the raw data against the themes identified to ensure accuracy. A series of follow up meetings were held with the various members of the research team (TA, SG, IR, SB) to further discuss and refine themes to ensure accuracy of the themes derived. Data extracted from included studies and other materials used for analysis are available on request.

Methodological quality of included studies was evaluated using the McMaster Critical Review Form: Qualitative Studies (Version 2.0).^44^ This tool includes nine categories that evaluate study purpose, literature, study design, sampling, data collection, procedural rigour, data analyses, overall rigour and conclusions and implications. A scoring system previously used by Guerin et al.^45^ was used to score studies out of a total score of 22, with positive criteria answered ‘yes’ scoring 1 point, and negative criteria answered ‘no’ or ‘not addressed’ scoring 0. The sum of scores was expressed as a percentage of applicable criteria met to allow for comparison of quality between studies. All studies were critically appraised by one reviewer (TA) and cross-checked for agreement by a second reviewer (SG).

In addition, two authors (TA, SG) used the Confidence in the Evidence from Reviews of Qualitative Research (CERQual) tool^46^ to assess confidence in the synthesised thematic findings of this review – that is, confidence that the findings of the review reflected the findings of the included studies. CERQual considers four components (the methodological limitations, the relevance, the coherence, and the adequacy of the data) to assess confidence (high, moderate, low, or very low) in each review finding. A high confidence rating indicates a high likelihood that the review finding reasonably represents the phenomenon of interest.^46^

## Results

**Figure 1.**
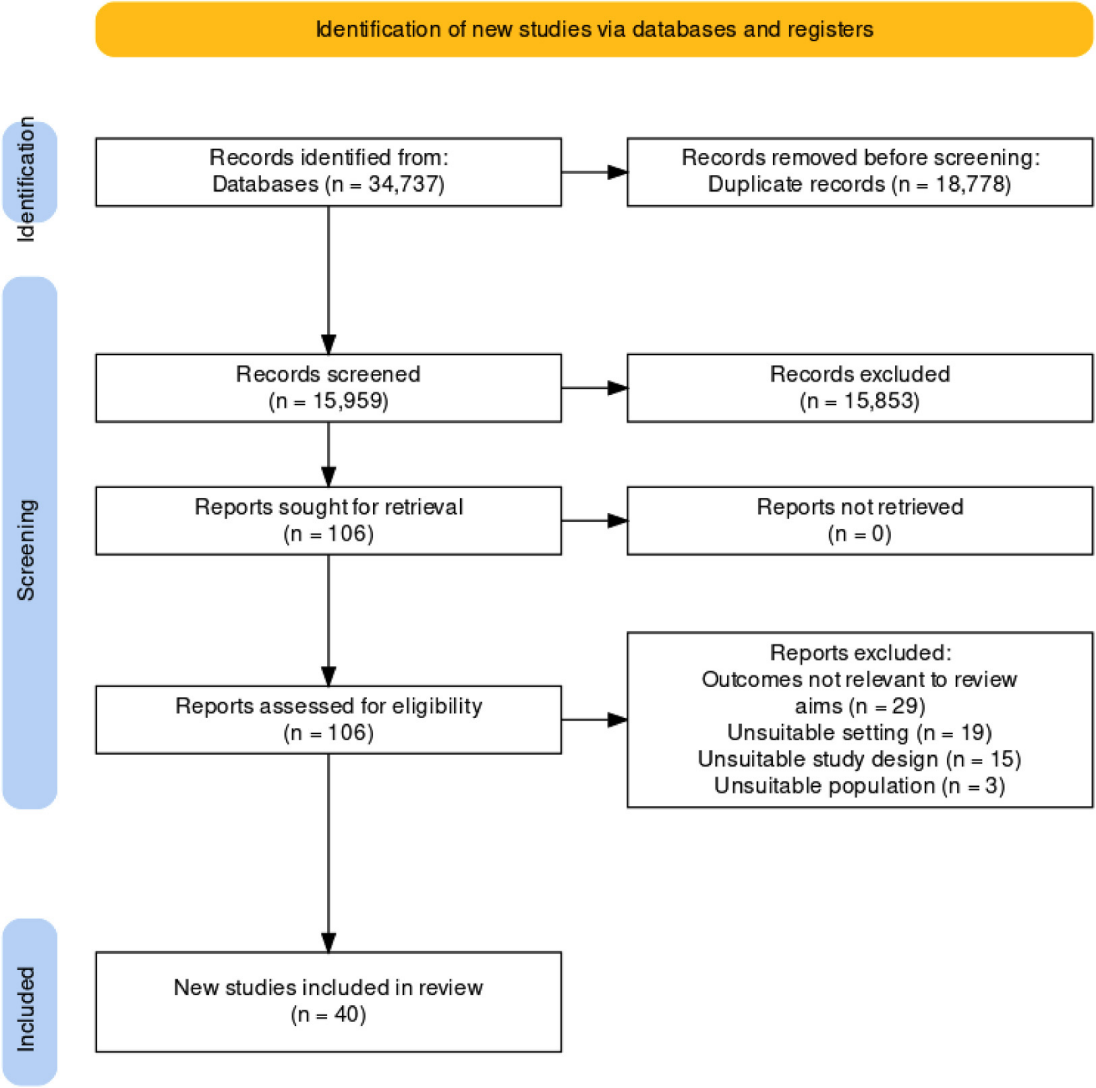
PRISMA flow diagram of search, screening, and selection process.^101^

The search identified 34,737 studies, with 18,778 duplicates removed leaving 15,959 studies screened at title/abstract level, with 106 studies progressing to full-text screening (see Figure 1 for the flow diagram of study selection). Forty studies were included in the review, with study characteristics for these presented in Table 1, and more detailed characteristics summarised in Supplemental File 3. Twenty-five of the studies explored perspectives from multiple disciplines,^31,47–70^ and the remaining 15 included participants from a single profession only.^71–85^ Overall, studies explored the perspectives of a combined 1408 health professionals working across acute hospitals,^31,48–52,54,57–59,61–65,67–74,76,78,80,82–84^ rehabilitation,^47,56,57,60,66,77^ mixed^53,55,75,79,81^ or other settings^85^ as nurses,^31,47–52,54–62,64–66,68–78,80,83,84^ physiotherapists,^31,48,50,51,53–57,59,60,62,64–66,68–70,79,85^ physicians or physician assistants^31,49,50,52,55–57,59,62–64,66–70,81,82^ and occupational therapists.^51,55–57,60,62,64–66,68^ Other professions included exercise therapy/sports scientists,^53^ mobility technicians/instructors,^58,68^ unspecified therapists,^47,78^ social and healthcare assistants,^55,65,68^ respiratory therapists,^66^ pharmacists,^68^ social workers,^56,57^ speech pathologists^56^ and other unspecified health professionals.^63,65^ No authors were required to be contacted for further data.

**Table 1.** Summary of included studies.

Authors	Country	Methods	Setting^a^	Participants
Annemans, 2022 ^47^	Belgium	Focus groups	Inpatient rehabilitation	*N* = 6, nurses and therapists
Boltz, 2011^71^	United States	Focus groups	Various wards	*N* = 55, nurses and patient care associates
Bor, 2022 ^48^	Netherlands	Semi-structured interviews	Medical oncology, haematology, cardiology and cardiothoracic surgery	*N* = 16, nurses, physical therapists and unit managers
Brown, 2007^49^	United States	Semi-structured interviews	Medical ward	*N* = 19, nurses and physicians
Chan, 2019^72^	Singapore	Focus groups	General hospital	*N* = 30, registered and enrolled nurses
De Klein, 2021^50^	Netherlands	Semi-structured interviews	Geriatrics and gastroenterology wards	*N* = 9, physiotherapists, nurses and doctors
Doherty-King, 2011^73^	United States	Semi-structured interviews	Adult medical and surgical wards	*N* = 25, registered nurses
Doherty-King, 2013^74^	United States	Semi-structured interviews	Adult medical or surgical wards	*N* = 25, registered nurses
Frederiksen, 2022^51^	Denmark	Focus groups	Geriatric and infectious and pulmonary medical diseases departments in a Danish hospital	*N* = 8, nurse assistants, nurses, physical therapists and occupational therapists
Geelen, 2021^52^	Netherlands	Semi-structured interviews and focus groups	Gastrointestinal, oncology surgery, internal medicine haematology, infectious diseases and cardiology wards	*N* = 30, nurses, nursing assistants, physician assistant and physicians
Geidl, 2019^53^	Germany	Focus groups	Various facilities	*N* = 58, department heads with a background in physiotherapy, exercise therapy/sports science or other
Gustafson, 2021^54^	United Kingdom	Focus group	General hospital	*N* = 4, physiotherapists and nurses
Hazra, 2023^55^	Canada	Online survey with qualitative component	Hospitals and healthcare centres	*N* = 338, physicians, nurses, physiotherapists, occupational therapists and healthcare aides
Hills, 2021^75^	Australia	Individual interviews	Inpatient subacute wards	*N* = 11, ward nurses and nurse leaders
Janssen, 2022 ^56^	Australia	Semi-structured interviews	Rehabilitation units (two mixed caseload, one neurological rehabilitation, one comprehensive stroke unit)	*N* = 22, nurses, nursing unit managers, rehabilitation physicians, physiotherapists, occupational therapists, social workers and speech pathologists
Jasper, 2023^57^	Australia	Focus group	Subacute geriatric ward and acute orthopaedic ward at a general hospital	*N* = 7, physiotherapists, occupational therapists, doctor, nurse and social worker
Johnson, 2019^58^	United States	Semi-structured interviews	General medicine ward	*N* = 8, physicians, mobility technician and nurses
King, 2016^76^	United States	Focus groups, one semi-structured interview	General medicine ward	*N* = 15, registered nurses and certified nursing assistants
Kirk, 2019^59^	Denmark	Go-along interviews during observation	Endocrinology, infectious diseases, and emergency departments	*N* = 79, nursing assistants, registered nurses, physiotherapists and physicians
Klooster, 2022^69^	The Netherlands	Semi-structured interviews	Cardiothoracic surgery, cardiology, pulmonary diseases, orthopaedics and traumatology, neurosurgery, abdominal surgery, urology, obstetrics and gynaecology, oral and maxillofacial surgery, cardiac care unit, and medical oncology wards	*N* = 15, physicians, physiotherapists, nurses and nurse assistants
Kneafsey, 2015^77^	United Kingdom	Semi-structured interviews	General rehabilitation ward, spinal unit and stroke rehabilitation ward	*N* = 33, care support workers, registered nurses and ward sisters
Kneafsey, 2013^60^	United Kingdom	Semi-structured interviews	General rehabilitation ward, spinal unit and stroke rehabilitation ward	*N* = 39, registered nurses, ward sisters, care support workers, therapy assistants, occupational therapists and physiotherapists
Koenders, 2020^31^	Netherlands	Semi-structured interviews	Cardiology and orthopaedics/trauma wards	*N* = 24, physical therapists, nurse assistants, nurses, physicians and physicians assistants
Lim, 2020a^78^	Singapore	Semi-structured interviews	General medical ward	*N* = 10, senior enrolled nurses, senior staff nurses, acting nurse clinician and nurse managers
Lim, 2020b^61^	United Kingdom	Focus groups	Acute medical wards for older people	*N* = 13, therapists (unspecified) and nurses
Lowe, 2018^79^	United Kingdom	Semi-structured interviews	General secondary care	*N* = 12, physiotherapists
Moore, 2014^62^	Canada	Focus groups	General hospital wards	*N* = 261, nurses, nurse practitioners, occupational therapists, physiotherapists, physicians, managers, other allied healthcare staff, and other unit staff such as personal support workers and ward clerks
Myers, 2021^63^	United Kingdom	Semi-structured interviews	Renal unit and complex medical unit	*N* = 8, sports and exercise medicine consultants and other healthcare professionals (unspecified)
Ohlsson-Nevo, 2020^80^	Sweden	Focus groups	Medical and surgical wards in three hospitals	*N* = 29, registered nurses and certified nursing assistants
Osinaike, 2021^81^	United Kingdom	Semi-structured interviews	Various; participants had spent at least 1 year in rotational foundation training.	*N* = 11, junior doctors
Pavon, 2021^64^	United States	Semi-structured interviews and focus groups	General medicine ward	*N* = 48, medical residents, nurses, certified nursing assistants, physical therapists and occupational therapists
Pedersen, 2020^82^	Denmark	Semi-structured interviews	Medical wards	*N* = 12, physicians
Pham, 2016^83^	Vietnam	Semi-structured interviews	Geriatrics, endocrinology, nephrology and urology departments	*N* = 25, nurses
Rasmussen, 2020^65^	Denmark	Focus groups	General hospital	*N* = 11, physiotherapists, occupational therapists, social and healthcare assistants, home-care workers and nurses
Scheerman, 2020^84^	The Netherlands	Semi-structured interviews	Internal medicine; traumatology; oncological surgery; and a combined ward of vascular surgery, nephrology and urology wards.	*N* = 51, nurses
van Dijk-Huisman, 2022^70^	The Netherlands	Semi-structured interviews	Combined university/tertiary internal medicine hospital wards	*N* = 16, nurses, physicians and physiotherapists
Williams, 2018^85^	United Kingdom/Ireland	Semi-structured interviews	Regional spinal cord injury centres	*N* = 18, physiotherapists
Wray, 2021^68^	United Kingdom	Focus groups	Geriatrics and combined orthogeriatric wards	*N* = 12, doctors, nurses, pharmacists, physiotherapists, occupational therapists, healthcare assistants and technical instructors
Wshah, 2021^66^	Canada	Semi-structured interviews	Pulmonary rehabilitation	*N* = 16, doctors, physiotherapists, occupational therapists, nurses and respiratory therapists
Zisberg, 2018^67^	Israel	Interviews and Focus Groups	Internal Medicine Wards	Unclear Total Sample Size, *N* = 116 Medical Professions However Multiphase Approach, Unclear Sample Size Of Qualitative Component.

^a^ Data only extracted when relevant to the settings meeting the eligibility criteria of this review.

The thematic analysis identified one central theme ‘*Physical activity is not a priority*’, and four subthemes*,* which are described in Table 2. Additional supporting quotes to the results can be found in Supplemental File 4. Our level of confidence in the review findings ranged from moderate to high and has been noted throughout the results. Table 3 includes the CERQual summary of qualitative findings.

**Table 2.** Central theme and subthemes with related key topics.

*Physical activity is not a priority*	*The hospital is a place for rest*	*A culture of bed rest*
		*Risk-reductive approach*
		*The physical environment*
	*There are not enough resources to make movement a priority*	*Not enough time, personnel or equipment*
		*High workloads*
		*Promoting physical activity is resource intensive, particularly in sick patients or patients with many attachments*
	*Everyone's job is no one's job*	*Shared responsibility but no clear perceived ownership*
		*Need for effective team collaboration*
		*Lack of knowledge/skills*
		*Patient’s own responsibility*
	*Policy and leadership drives priorities*	*Falls prevention, manual handling and mobility policies can deter from physical activity promotion*
		*Leadership in promotion of physical activity was important*

**Table 3.** CERQual summary of qualitative findings.

				CERQual	
Main theme	Subthemes	Summary	Studies	Confidence	Explanation
Physical activity is not a priority	The hospital is a place for rest	The hospital was often seen to facilitate a culture of bed rest through the physical environment, staff, and systems, with favouritism towards a risk-reductive approach to care delivery.	^31,47–50,52,54,56,57,59,60,62–65,68–75,77,78,80,82–84^	High confidence	Twenty-nine studies contribute to this finding. There were minor concerns about methodological limitations and coherence of those studies. There were no/very minor concerns about adequacy and relevance.
	There are not enough resources to make movement a priority	Health professionals felt that promotion of movement could be resource intensive in an already resource-limited (specifically lacking in time, personnel, or equipment), high workload setting. Subsequently, resources were allocated to other priority areas.	^31,48–52,54–56,58–64,66,68,70–75,78–80,82–85^	High confidence	Thirty-one studies contribute to this finding. There were minor concerns about methodological limitations and relevance, and no/very minor concerns about coherence and adequacy of data.
	Everyone's job is no one's job	Health professionals perceived there to be a shared responsibility, yet noted a lack of ownership, in supporting patient movement amongst the interdisciplinary team and the patient themselves. Reduced knowledge and skills amongst some team members was noted as a barrier to supporting movement.	^31,48,50–53,55,56,59–72,74,75,80–85^	Moderate confidence	Thirty studies contribute to this finding, with minor concerns with methodological limitations, coherence, adequacy, and relevance.
	Policy and leadership drives priorities	Health professionals practice, perspectives and prioritisation of movement support was influenced by policies and leaders. Falls prevention, manual handling and mobility-related policies prioritised safety over movement further contributing to a risk reductive approach	^48,56,59–64,67–69,71–73,76,77^	High confidence	Sixteen studies contribute to this finding. There were no/very minor concerns about methodological limitations, coherence, adequacy, and relevance.

### Central theme: physical activity is not a priority

Health professionals frequently reported that they were aware of the benefits of physical activity promotion^31,59,63,64,66,68,69,76,78,80–82,84,85^ and the importance of optimising inpatient movement behaviour to prevent complications^60,63,64,68–71,74,80,83,84^ or functional decline,^64,68,70,72,76,78,84^ and to optimise recovery.^64,69,72,80^ They noted that the inpatient setting could be an opportunity to instil education and to promote long-term positive physical activity behaviour to patients,^63,82,85^ and that increasing physical activity during a patient's admission could improve other aspects of the patient's wellbeing and their general experience in hospital^56,69,75,80^:

‘*It's staggering how much time they [patients] spend alone. There's a potential connection here. … If we tackle the boredom, we tackle the sedentary behaviour, there is a link, and we will solve the social isolation. …*’ – Nurse leader^75^

However, despite strong statements that physical activity is critical, optimising movement behaviours was overall not seen as a priority in the inpatient setting over other tasks.^49–51,59,61,70,74,75,79,82,84^ The subthemes showed a range of contributing, often interrelated, factors that reinforced that physical activity was not a priority, and also demonstrated potential contributing factors that resulted in this lack of prioritisation.

### Subtheme 1: ‘The hospital is a place for rest’

First, health professionals perceived that unit expectations and culture influenced how staff facilitated physical activity and/or discouraged sedentary time, with a culture that often reinforced the hospital as a place of rest (CERQual assessment: high confidence).^48,50,64,69,71,73,74,76,80^ This culture was often not conducive to physical activity, with a culture of bed rest (whereby some patients associated hospitalisation with rest)^50,52,59,60,62,65,70,71,82–84^ contributing to this concept of physical activity not being a priority:

‘*Many of the older patients do not want to get out of bed. It seems that they were raised to believe that when you are ill, you must recover in bed*’ *–* Nurse assistant^59^

This bed rest culture and pacification of the patient was described as being influenced by families or caregivers, who could act as a barrier^71,72,75,80^ or an enabler to physical activity.^49,52,56,57,71,72,75,80^ Health professionals also perceived that some patients lacked motivation to move during hospitalisation,^31,49,50,59,62,65,72,83,84^ (particularly when patients were elderly^49,50,59,80,82–84^), which may further reinforce bed rest culture:

‘*I just think he is older now, and he is not as motivated as younger people are, and he has been through so much. I just don’t think he wants to do it.*’ – Unknown profession^49^

A perceived fear of falling further influences this, where health professionals felt patients’ fear of falling discouraged them from moving more^60,62,68,71,72^:

‘*They may have had a fall at home that warranted this admission. Consequently, the phobia is there as they refuse to get out of bed due to the fear of falling again.*’ – Nurse^72^

These fears were said to be exacerbated by health professionals who provide constant assistance, reinforcing the lack of confidence patients have in their mobility and thereby further reinforcing a culture of rest^68^:

‘*…if we’re like mobilising patients and we’re clinging on to them like that, it does nothing for anyone's confidence, you know, them thinking I need someone to be on my hip the whole time, rather than if you just take a step back and you know.*’ – Unknown profession^68^

Subsequently, this culture was perceived to be reinforced by the preference for and reinforcement of a risk-reductive approach, where falls prevention was prioritised over patient physical activity^47,49,56,60,64,68,72,77^:

‘*I think a big part of it is safety…part of the culture has become that the room seems like the safest place for the patient … … keep them in bed.*’ – Physiotherapist^56^

Health professionals perceived that the physical environment of the hospital reflected this culture of bed rest^31,48,49,52,56,57,62,70,78,80,82,83^; cited as promoting sedentary time and lacking in features to incentivise physical activity.^48,56,70,82^ The hospital environment was said to lack space for physical activity^,59,62,70,71,78,82,83^or available space was not fit for purpose,^56^ appealing^48,56^ or known to patients that it was available for their use.^56^ The hospital room and bed space in particular was thought of as somewhat of a centrepiece, where its design and layout promoted patients to spend time in bed^31,49,52,57,70,82^:

‘*No, when the room is organized around the bed, and everything is within reach and the television is also free; which means it's available for everyone; then it's incredibly tempting for people to stay in their beds.*’ – Nurse^52^

Health professionals felt patients might engage in more physical activity if the ward environment was better designed to facilitate this,^47,49,57,59,84^ and in particular if the environment made patients feel safe^57,82^:

‘*If you walk badly and are a little dizzy and so on, it's not interesting to walk in a hallway which is narrow and where people come rushing. Then I think you prefer staying in bed. There you’ll feel safe.*’ – Physician^82^

However, this culture of bed rest is perhaps not entirely unsubstantiated given in some cases, medical bed rest was indeed believed to be indicated. Health professionals were also clear that physical activity was not always an appropriate priority for all patients in the hospital setting during admission^49,50,54,63,64,72,73,80,82^ due to some patients being too unwell,^50,54,64,70,72,73,82^ in too much pain^50,54,64,70,72,73,82^ and/or limited in their capacity to move because of medical attachments.^49,50,54,63,64,70,72,73,80,82^

Taken together, the hospital was perceived as being a place for rest, where risk reduction was prioritised over physical activity, and so physical activity was subsequently not a priority in the inpatient setting.

### Subtheme 2: ‘There are not enough resources to make movement a priority’

Despite strong beliefs overall that physical activity and minimising sedentary behaviour was important, staff most frequently cited that resource constraints were a barrier to promoting increased physical activity and reduced sedentary behaviour of their patients (CERQual assessment: high confidence).^31,48–52,54–56,58–64,66,68–75,78–80,82–85^ Many health professionals described a lack of time,^31,49–52,56,60–62,66,68–72,75,79,80,82^ personnel/staff^49,51,54–56,58,60,61,63,68,70,71,74,75,78,80,84,85^ and equipment.^49,52,54,55,59,62,64,70,73,83^ This was challenged further in the context of high workloads^48,62,70,72,75,80,82,84^:

‘*…sometimes it is due to a high workload. You know it is important and benefits the patient, but you don't have enough time unfortunately… when other things have to be done, it is not a priority.*’ – Nurse^84^

Health professionals felt physical activity would be improved and/or sedentary time reduced if there were more staff available to promote these behaviours.^51,55,56,62,73,74^ Additionally, patients who were sicker, had more attachments and/or required assistance to mobilise^49,54,56,70^ were perceived to increase workloads^49,72,74,75^ in this already resource-stretched environment, leading to health professionals being less inclined to facilitate physical activity, and/or patients to feel restricted to move:

‘*I hate to say it, but I think on some days, it does [affect mobility]. You have patients who have TPN [total parenteral nutrition] and blood and Foleys and chest tubes, you are probably less likely to [get people out of bed].*’ – Nurse^49^

Given facilitation of physical activity is considered resource intensive,^60,68,75^ health professionals sometimes compensated for a lack of resources (particularly when workloads were high^50,59,74,84^) by increasing the assistance provided beyond what was necessary in the interest of saving time^60,68,70,72,80^:

‘*Things can be done faster if we provide total assistance. That's why we always helped them more than we should.*’ – Nurse^72^

A lack of resources (of time, personnel, and equipment) appeared, in some cases, to lead to not only an omission of promotion of optimal movement behaviours, but active obstruction to incidental movement as well.

### Subtheme 3: ‘Everyone's job is no one's job’

There were conflicting views amongst health professionals regarding roles and responsibilities for promoting or facilitating physical activity and reducing sedentary behaviour. This indicates that perhaps these behaviours are not prioritised by members of the team due to the perception of a shared responsibility^48,55,56,62,64,67,70,71,74,81,82,84,85^ without effective clarification of roles in assessment and promotion of physical activity (CERQual assessment: moderate confidence). Although health professionals felt information and education should be provided to patients,^55,57,58,61,66,70,71,83,85^ they perceived their own reduced knowledge and skills as a barrier to promoting physical activity,^50,54,55,62,64,66,70,73,75,83,85^ perceiving this as a reason for why they attributed responsibility to others^50,59,82,83^:

‘*I tell them they should get out of bed. But not like, “walk the stairs” or something. I don’t know what to advise them. I leave that to the physiotherapist.*’ – Physician^50^

In addition to this, health professionals felt a lack of knowledge of a patient's baseline mobility^61,62,71^ and/or current mobility status^51,62,69–71^ as barriers to facilitating physical activity. There were conflicting opinions as to which profession/s are responsible and/or capable of collecting and/or assessing this. Physiotherapists were frequently perceived as responsible for mobility assessment,^80,82^ with physicians^84^ and nurses^67^ reported as also being capable. This information was perceived as important for setting expectations for inpatient physical activity and could influence how health professionals promoted activity amongst different patients^64,71,73^:

‘*We don’t know what the patient can do. Often, older patients look very frail. We don't know if they can stand, for instance.*’ – Patient Care Associate^71^

In terms of promotion of physical activity, physiotherapists^51,52,59,60,64,70,80,82,84,85^ and nurses^50,52,67,72,75,80,82–84^ were commonly described as the most appropriate health professionals to do so:

*‘I think we play a pretty important role, we are with our patients every day and we have the time to get in there and encourage the exercises that [the] physio suggested.*’ – Nurse^75^

However, physicians were also noted by health professionals as having an important role in reinforcing these behaviours to patients,^52,57,59,63,70,80,82^ and it was perceived that patients would be more likely to listen to physicians over other members of the team^59,70,80,82^:

‘*And even the doctors – sometimes we have to ask the physician doing the round, “Please, tell this patient how important it is that he gets up and moves around a bit.” Sometimes it helps and it's like they need that.*’ – Nurse^80^

Interestingly, health professionals also noted that activity was not always their responsibility as patients held an element of ownership over their movement behaviours and recovery^31,47,50,52,53,80,84^:

‘*Well, I think: yeah right, I am not a cop. If someone really does not want to get out of bed because he feels to sick or does not want to. Well, at some point it is just the responsibility of the patient.*’ – Nurse^50^

However, it was also acknowledged that personal responsibility could be restricted in the inpatient environment^80^:

‘*Suddenly, you find yourself in a hospital and you can’t really take any personal responsibility – you’re at the mercy of other people who make all the decisions*’ – Certified Nursing Assistant^80^

Subsequently, staff also acknowledged the need to take responsibility for making patients aware of the importance of increasing physical activity and reducing sedentary behaviour during their admission,^50,62,71,82,84^ as well as what they are expected to^50,52^ and are allowed to do^68,82^:

‘*We (sic) probably maybe better at promoting what not to do rather than what you can do*.’ – Profession unknown^68^

Despite some uncertainty around key roles and responsibilities, health professionals felt there was a shared responsibility to facilitate these behaviours^50–52,55,56,63,66,67,70,71,75,80,82^ and a need for a team-based approach^48,53,63,65,66,68–72,75,80^ utilising interprofessional collaboration to effectively facilitate movement behaviours in this setting^48,51,53,55,59,63,65,66,68–72,75^:

‘*Getting people out of bed and moving can’t happen if everyone doesn’t help.*’ – Patient care associate^71^

If this collaboration was poor (which may result in the presence of skewed roles and responsibilities), it was thought to compromise effective facilitation of these behaviours.^59,62^ Conflicting and unclear roles and responsibilities around the promotion of physical activity in this setting is likely to contribute to this lack of prioritisation of physical activity – where no clear ownership exists.

### Subtheme 4: ‘Policy and leadership drives priorities’

Finally, health professionals noted the influence of policies and leaders and that these ultimately trickled down to how their own day-to-day tasks were prioritised (CERQual assessment: high confidence). Falls prevention, manual handling and/or other mobility-related policies were found to promote a risk reductive approach that prioritised safety over movement, thereby reinforcing a culture of bed rest and acting as a barrier to optimising physical activity and reducing sedentary behaviour.^56,60–62,64,68,71–73,77^ This was reinforced by other related barriers such as organisational quality indicators,^60,67,68,72^ with health professionals expressing a fear of patients falling or injuring themselves while moving,^47,49,56,60–62,64,68,71–73,77^ and concerns about sustaining work related injuries while assisting patients^60,62,73^:

‘*While they are in bed they are not giving trouble to anybody. It is less work and, second, because of liability issues in terms of patients falling and hurting themselves while they are in the hospital. I think everybody is very concerned with that, but I think mainly because it is less work.*’ – Physician^49^

Beyond those related to falls, additional hospital policies were perceived as restrictive to the capacity of health professionals to promote patient physical activity.^59,60,67,71,77^ These included manual handling policies that favoured passive patient transport^60,71,77^ and policies that required physician and/or physiotherapy clearance before patients were allowed to be mobilised.^71,77^ Health professionals reported they felt hesitant to facilitate physical activity of their patients in the interest of safety, as well as due to a fear of repercussions should policy or quality indicators be compromised.^60,67,68,72^

‘*I walk a lot of steps during the day because the patients call for more coffee, which I have to pick up in the corridor where we have placed the coffee machine for self-supporting patients and their relatives or they want me to change their sheets. It is interesting because a lot of the patients do it by themselves at home*’ – Nurse ^59^

Additionally, health professionals reported that leadership support from senior clinicians and/or management was important to enable and motivate staff to promote physical activity^48,63,69,71,76^:

‘*On my unit, preventing physical decline is an important goal. We review functional status during rounds, and everyone sees this as a priority. I think this is because the leadership here believes that older people deserve to stay functional.*’ – Nurse^71^

This demonstrates that health professionals may look to policies and leadership in developing their priorities in clinical practice, and thus contribute to the overarching theme of physical activity not being a priority in the inpatient setting.

### Study quality and reporting

The methodological quality of the included studies in this review was variable, with critical appraisal scores ranging from 36 to 95%. Consensus agreement was reached by two reviewers. Common methodological issues related to a lack of clearly defined theoretical perspective, not mentioning whether data collection continued until thematic saturation, limited disclosure of the role of the researcher and/or relationship with participants and a lack of disclosure of identified assumptions and biases of researcher.

## Discussion

The aim of this systematic review was to explore the perspectives of health professionals on physical activity and sedentary behaviour of hospitalised adults, using an extensive search strategy, to understand factors that may contribute to these behaviours in patients in the inpatient environment. The review findings, which synthesise findings from 40 studies inclusive of over 1408 health professionals, indicate that despite knowing the benefits, physical activity is not perceived as a priority in inpatient settings by health professionals. Contributing factors are multifactorial and occur across multiple levels of the healthcare system. This builds on findings from similar related reviews^35,36,86^ and suggests current physical activity interventions are not translatable to the hospital setting.^87^ In particular, this review highlights the influence of prioritisation on the provision of physical activity and sedentary behaviour change support in this setting, notably, that health professionals recognise the benefits of movement for their patients but are met with challenges in managing competing demands.

There was a tendency to prioritise risk reduction over physical activity. This approach contributes to a culture of bed rest to promote safety that aligns with an outdated biomedical model of care.^36,86^ Physical activity was perceived to increase falls risk and unsuitable for patients who were unwell or older in age,^36,38,86^ resulting in avoidance.^88,89^ However, increased physical activity is associated with improved function,^88^ reduced falls risk ^89^ and shorter length of stay.^90^ Importantly, these improvements are not associated with an increase in falls.^89^ The priority for safety and risk reduction should be acknowledged when designing interventions for this setting, while falls prevention policies could also explore ways of ensuring physical activity is not unnecessarily restricted during implementation.

Inconsistencies in how physical activity is defined by health professionals,^31,52,67,79^ together with the absence of clear guidelines for physical activity in hospitalised adults, may create uncertainty on how to promote these behaviours in this setting. To date, there is only one Delphi study that recommends increased physical activity and reduced sedentary time in hospitalised older adults.^91^ Strategies for implementation are unclear and what targets should be aimed for in other hospitalised populations (e.g. rehabilitation). Further, health professionals often describe physical activity being accumulated through structured therapy when in hospital,^36,48,60^ rather than identifying opportunities such as transfers, bed mobility, walking and showering as potentially beneficial incidental activity and opportunities to break up sitting time.^92^ These incidental opportunities may also have health benefits without the same resource requirements as structured therapy, and could be a feasible way to address this problem of low activity and high sedentary behaviour observed in hospitalised adults. Clear definitions of strategies used to increase activity in the hospital setting will be important.

Hospital resourcing, processes and environments may not be conducive to support patients to move more and sit less. Resource constraints are commonly reported by health professionals as barriers to physical activity interventions. Identifying the potential benefits at a system level may assist in resource allocation within organisations for staff and time to invest in these interventions. For example, interventions to increase physical activity during acute hospitalisation has shown cost savings of up to $300 USD per patient, per hospital stay.^93^ Considering this cost-benefit during planning, prioritisation and resourcing will be important in future hospital design. Further, processes such as meals being brought to the bedside^52^ and environments with limited communal spaces and dining rooms^94,95^ reduce opportunities for activity and encourage patients to remain in bed, accumulating high volume of sedentary behaviour. These should be considered in future hospital design.^21,95–98^

This review found that health professionals perceived patients to have low motivation to be physically active.^31,49,50,59,62,65,72,83,84^ However, previous literature has disputed this, with patients reporting that health professionals do not value mobility, are not interested in it and/or find it burdensome to assist.^36,49,86^ Hospitalised adults express a desire to engage in meaningful physical activity,^31,99,100^ value the encouragement from health professionals and feel inactivity exacerbates feelings of a loss of freedom during hospitalisation.^31^ Improving physical activity has the potential to induce not only physical benefits, but also help patients feel ‘liberated from the bed and more independent’,^31^ regaining a sense of autonomy and reducing boredom.^36^

This review has several strengths. Firstly, we used a comprehensive search strategy and to our knowledge this is the largest qualitative review on this topic. Secondly, the research team has combined experience in qualitative research, physical activity, and clinical experience in hospital and rehabilitation settings. A limitation of this review was that qualitative work embedded within interventional studies were included,^48,54,61–63,67,76,82^ so findings from these studies may have been influenced by participant involvement with an intervention. A single author extracted data; however, this was cross-checked by a second author with conflicts resolved by a third author. Our inclusion criteria of studies published in English subjects this research to potential publication and English language bias, and this should be considered when interpreting the findings of this research. This review was undertaken during the COVID-19 pandemic, and although we excluded COVID-19 specific studies, it is important to note that most included studies explored this phenomenon pre-COVID-19. Factors such as isolation protocols, visitor restrictions and staffing restraints have likely impacted on inpatient physical activity and sedentary behaviour. Finally, the included studies ranged in quality, and we did not exclude low-quality studies from the analysis. However, we did not find grossly different conclusions in low-quality studies and would not expect excluding based on quality to have changed the findings of this review.

In conclusion, physical activity is not a priority in the hospital setting. Instead, the hospital is often considered a place for rest, and encourages a risk reductive approach that can promote immobility despite the evidenced benefits of physical activity and risks of inactivity and high sedentary time. Multilevel challenges with resources, role clarification, a lack of clear leadership and policies that foster bed rest culture and immobility must be addressed to comprehensively enable delivery of evidence-based, person-centred care.

**Clinical messages**

- Physical activity is not a priority in the inpatient setting despite the evidenced benefits.
- Multilevel considerations are required in developing interventions to target these behaviours.
- An interdisciplinary teams-based approach is recommended, however, requires supportive clinical environments that provide the appropriate resources, leadership, and policy support to promote cultural change.

## Supplemental Material

sj-docx-1-cre-10.1177_02692155231170451 - Supplemental material for Perspectives of health professionals on physical activity and sedentary behaviour in hospitalised adults: A systematic review and thematic synthesis Click here for additional data file. Supplemental material, sj-docx-1-cre-10.1177_02692155231170451 for Perspectives of health professionals on physical activity and sedentary behaviour in hospitalised adults: A systematic review and thematic synthesis by Tahlia Alsop, James Woodforde, Ingrid Rosbergen, Niruthikha Mahendran, Sandra Brauer, and Sjaan Gomersall in Clinical Rehabilitation
