# Supplementary material for: Perspectives of health professionals on physical activity and sedentary behaviour in hospitalised adults: A systematic review and thematic synthesis
Source: Clin Rehabil. 2023 Apr 17;37(10):1386–405. doi: 10.1177/02692155231170451 (PMC10426259; doi:10.1177/02692155231170451)
Supplement: sj-docx-1-cre-10.1177_02692155231170451 - Supplemental material for Perspectives of health professionals on physical activity and sedentary behaviour in hospitalised adults: A systematic review and thematic synthesis [file sj-docx-1-cre-10.1177_02692155231170451.docx]

**Supplemental files**

*Supplemental File 1:* PRISMA checklist

*Supplemental File 2:* Search strategy

*Supplemental File 3:* Expanded study characteristics table

*Supplemental File 4:* Additional supporting quotes

*Supplemental File 5:* Quality appraisal results

**Supplemental File 1:** PRISMA Checklist

| **Section and Topic** | **Item #** | **Checklist item** | **Location where item is reported** |
| --- | --- | --- | --- |
| **TITLE** | | |  |
| Title | 1 | Identify the report as a systematic review. | Separate title page file |
| **ABSTRACT** | | |  |
| Abstract | 2 | See the PRISMA 2020 for Abstracts checklist. | Separate abstract file |
| **INTRODUCTION** | | |  |
| Rationale | 3 | Describe the rationale for the review in the context of existing knowledge. | Introduction |
| Objectives | 4 | Provide an explicit statement of the objective(s) or question(s) the review addresses. | Introduction |
| **METHODS** | | |  |
| Eligibility criteria | 5 | Specify the inclusion and exclusion criteria for the review and how studies were grouped for the syntheses. | Methods |
| Information sources | 6 | Specify all databases, registers, websites, organisations, reference lists and other sources searched or consulted to identify studies. Specify the date when each source was last searched or consulted. | Methods |
| Search strategy | 7 | Present the full search strategies for all databases, registers and websites, including any filters and limits used. | Methods |
| Selection process | 8 | Specify the methods used to decide whether a study met the inclusion criteria of the review, including how many reviewers screened each record and each report retrieved, whether they worked independently, and if applicable, details of automation tools used in the process. | Methods |
| Data collection process | 9 | Specify the methods used to collect data from reports, including how many reviewers collected data from each report, whether they worked independently, any processes for obtaining or confirming data from study investigators, and if applicable, details of automation tools used in the process. | Methods |
| Data items | 10a | List and define all outcomes for which data were sought. Specify whether all results that were compatible with each outcome domain in each study were sought (e.g. for all measures, time points, analyses), and if not, the methods used to decide which results to collect. | Methods |
|  | 10b | List and define all other variables for which data were sought (e.g. participant and intervention characteristics, funding sources). Describe any assumptions made about any missing or unclear information. | Methods |
| Study risk of bias assessment | 11 | Specify the methods used to assess risk of bias in the included studies, including details of the tool(s) used, how many reviewers assessed each study and whether they worked independently, and if applicable, details of automation tools used in the process. | Methods |
| Effect measures | 12 | Specify for each outcome the effect measure(s) (e.g. risk ratio, mean difference) used in the synthesis or presentation of results. | N/A |
| Synthesis methods | 13a | Describe the processes used to decide which studies were eligible for each synthesis (e.g. tabulating the study intervention characteristics and comparing against the planned groups for each synthesis (item #5)). | N/A |
|  | 13b | Describe any methods required to prepare the data for presentation or synthesis, such as handling of missing summary statistics, or data conversions. | N/A |
|  | 13c | Describe any methods used to tabulate or visually display results of individual studies and syntheses. | Methods |
|  | 13d | Describe any methods used to synthesize results and provide a rationale for the choice(s). If meta-analysis was performed, describe the model(s), method(s) to identify the presence and extent of statistical heterogeneity, and software package(s) used. | Methods |
|  | 13e | Describe any methods used to explore possible causes of heterogeneity among study results (e.g. subgroup analysis, meta-regression). | N/A |
|  | 13f | Describe any sensitivity analyses conducted to assess robustness of the synthesized results. | N/A |
| Reporting bias assessment | 14 | Describe any methods used to assess risk of bias due to missing results in a synthesis (arising from reporting biases). | Methods |
| Certainty assessment | 15 | Describe any methods used to assess certainty (or confidence) in the body of evidence for an outcome. | Methods |
| **RESULTS** | | |  |
| Study selection | 16a | Describe the results of the search and selection process, from the number of records identified in the search to the number of studies included in the review, ideally using a flow diagram. | Results |
|  | 16b | Cite studies that might appear to meet the inclusion criteria, but which were excluded, and explain why they were excluded. | Results |
| Study characteristics | 17 | Cite each included study and present its characteristics. | Results |
| Risk of bias in studies | 18 | Present assessments of risk of bias for each included study. | Results |
| Results of individual studies | 19 | For all outcomes, present, for each study: (a) summary statistics for each group (where appropriate) and (b) an effect estimate and its precision (e.g. confidence/credible interval), ideally using structured tables or plots. | N/A |
| Results of syntheses | 20a | For each synthesis, briefly summarise the characteristics and risk of bias among contributing studies. | Results |
|  | 20b | Present results of all statistical syntheses conducted. If meta-analysis was done, present for each the summary estimate and its precision (e.g. confidence/credible interval) and measures of statistical heterogeneity. If comparing groups, describe the direction of the effect. | N/A |
|  | 20c | Present results of all investigations of possible causes of heterogeneity among study results. | Results |
|  | 20d | Present results of all sensitivity analyses conducted to assess the robustness of the synthesized results. | N/A |
| Reporting biases | 21 | Present assessments of risk of bias due to missing results (arising from reporting biases) for each synthesis assessed. | Results |
| Certainty of evidence | 22 | Present assessments of certainty (or confidence) in the body of evidence for each outcome assessed. | Results |
| **DISCUSSION** | | |  |
| Discussion | 23a | Provide a general interpretation of the results in the context of other evidence. | Discussion |
|  | 23b | Discuss any limitations of the evidence included in the review. | Discussion |
|  | 23c | Discuss any limitations of the review processes used. | Discussion |
|  | 23d | Discuss implications of the results for practice, policy, and future research. | Discussion |
| **OTHER INFORMATION** | | |  |
| Registration and protocol | 24a | Provide registration information for the review, including register name and registration number, or state that the review was not registered. | Methods |
|  | 24b | Indicate where the review protocol can be accessed, or state that a protocol was not prepared. | Methods |
|  | 24c | Describe and explain any amendments to information provided at registration or in the protocol. | Methods |
| Support | 25 | Describe sources of financial or non-financial support for the review, and the role of the funders or sponsors in the review. | Funding |
| Competing interests | 26 | Declare any competing interests of review authors. | Declaration of competing interests |
| Availability of data, code and other materials | 27 | Report which of the following are publicly available and where they can be found: template data collection forms; data extracted from included studies; data used for all analyses; analytic code; any other materials used in the review. | Methods |

*From:*  Page MJ, McKenzie JE, Bossuyt PM, Boutron I, Hoffmann TC, Mulrow CD, et al. The PRISMA 2020 statement: an updated guideline for reporting systematic reviews. BMJ 2021;372:n71. doi: 10.1136/bmj.n71

**Supplemental File 2:** Search terms

**Original search date:** 20^th^ May 2021

**Updated search:** 2^th^ March 2023

| **PubMed** |
| --- |
| (((("Qualitative Research"[MeSH Terms] OR "qualitative"[All Fields] OR "qualitatively"[All Fields] OR "focus group"[All Fields] OR "focus groups"[All Fields] OR "interview*"[All Fields] OR "attitudes"[All Fields] OR "perspective*"[All Fields] OR "perception*"[All Fields]) AND ("sedentary behavior"[MeSH Terms] OR ("sedentary"[All Fields] AND "behavior"[All Fields]) OR "sedentary behavior"[All Fields] OR "physical activity"[All Fields] OR "sitting time"[All Fields] OR "sitting"[All Fields] OR ("walked"[All Fields] OR "walking"[MeSH Terms] OR "walking"[All Fields] OR "walks"[All Fields]) OR "exercising"[All Fields] OR "exercise"[All Fields] OR "exercise"[MeSH Terms] OR ("sedentary"[Title/Abstract] AND "behaviour"[Title/Abstract]) OR "sedentary behaviour"[All Fields] OR "inactivity"[All Fields] OR "mobility"[Title/Abstract]) AND (((((((((("acute care"[All Fields] OR "hospital*"[Title/Abstract] OR "hospitalization"[All Fields] OR ("hospital s"[All Fields] OR "hospitalisation"[All Fields] OR "hospitalization"[MeSH Terms] OR "hospitalization"[All Fields] OR "hospitalised"[All Fields] OR "hospitalising"[All Fields] OR "hospitality"[All Fields] OR "hospitalisations"[All Fields] OR "hospitalizations"[All Fields] OR "hospitalize"[All Fields] OR "hospitalized"[All Fields] OR "hospitalizing"[All Fields] OR "hospitals"[MeSH Terms] OR "hospitals"[All Fields] OR "hospital"[All Fields]) OR ("hospital s"[All Fields] OR "hospitalisation"[All Fields] OR "hospitalization"[MeSH Terms] OR "hospitalization"[All Fields] OR "hospitalised"[All Fields] OR "hospitalising"[All Fields] OR "hospitality"[All Fields] OR "hospitalisations"[All Fields] OR "hospitalizations"[All Fields] OR "hospitalize"[All Fields] OR "hospitalized"[All Fields] OR "hospitalizing"[All Fields] OR "hospitals"[MeSH Terms] OR "hospitals"[All Fields] OR "hospital"[All Fields]) OR "acute care"[All Fields] OR "rehabilitation"[MeSH Terms] OR "rehabilitation"[All Fields] OR "rehabilitation"[MeSH Subheading]) NOT "paediatric*"[All Fields]) NOT "pediatric*"[All Fields]) NOT "mental health unit"[All Fields]) NOT "intensive care unit"[All Fields]) NOT "COVID-19"[All Fields]) NOT "coronavirus"[All Fields]) NOT "cancer care"[All Fields]) NOT "oncology"[All Fields]) NOT "palliative"[All Fields]) AND ("health personnel"[MeSH Terms] OR "health care personnel"[All Fields] OR "health personnel"[All Fields] OR "health professionals"[All Fields] OR "health professional"[All Fields] OR "health care professional"[All Fields] OR "health care professionals"[All Fields] OR "healthcare professionals"[All Fields] OR "healthcare professional"[All Fields] OR "healthcare personnel"[All Fields] OR "allied health personnel"[All Fields] OR "allied health personnel"[MeSH Terms] OR "physiotherapist*"[All Fields] OR "physical therapists"[MeSH Terms] OR "physical therapist*"[All Fields] OR "occupational therapists"[MeSH Terms] OR "occupational therapists"[All Fields] OR "occupational therapist"[All Fields] OR "physical therapy modalities"[MeSH Terms] OR "physical therapy modalities"[All Fields] OR "physiotherapy"[All Fields] OR "physiotherapies"[All Fields] OR "physical therapy"[All Fields] OR "occupational therapy"[MeSH Terms] OR "occupational therapy"[All Fields] OR "nurse s"[All Fields] OR "nurses"[MeSH Terms] OR "nurses"[All Fields] OR "nurse"[All Fields] OR "nurses s"[All Fields] OR "Nursing"[MeSH Terms] OR "physicians"[MeSH Terms] OR "physicians"[All Fields] OR "doctor"[All Fields] OR "doctors"[All Fields] OR "exercise physiologist"[All Fields] OR "interdisciplinary studies"[MeSH Terms] OR "multidisciplinary"[All Fields] OR "multidiscipli*"[All Fields] OR "clinician*"[All Fields])) NOT "Review"[Publication Type]) AND "english"[Language]) AND ((journalarticle[Filter]) AND (english[Filter])) |
| **Embase** |
| ('health care personnel'/exp OR 'health care personnel' OR 'health personnel'/exp OR 'health personnel' OR 'health professionals' OR 'health professional'/exp OR 'health professional' OR 'health care professional'/exp OR 'health care professional' OR 'health care professionals' OR 'healthcare professionals' OR 'healthcare professional'/exp OR 'healthcare professional' OR 'healthcare personnel'/exp OR 'healthcare personnel' OR 'allied health personnel'/exp OR 'allied health personnel' OR physiotherapist* OR 'physical therapists'/exp OR 'physical therapists' OR 'physical therapist*' OR 'occupational therapists'/exp OR 'occupational therapists' OR 'occupational therapist'/exp OR 'occupational therapist' OR 'physical therapy modalities'/exp OR 'physical therapy modalities' OR 'physiotherapy'/exp OR 'physiotherapy' OR 'physiotherapies' OR 'physical therapy'/exp OR 'physical therapy' OR 'occupational therapy'/exp OR 'occupational therapy' OR 'nurse s' OR 'nurses'/exp OR 'nurses' OR 'nurse'/exp OR 'nurse' OR 'nurses s' OR 'nursing'/exp OR 'nursing' OR 'physicians'/exp OR 'physicians' OR 'doctor'/exp OR 'doctor' OR 'doctors' OR 'exercise physiologist' OR 'interdisciplinary studies'/exp OR 'interdisciplinary studies' OR 'multidisciplinary' OR multidiscipli* OR 'clinician'/exp OR 'clinician') AND (('hospital':ab OR 'hospital':ti OR 'acute care'/exp OR 'acute care' OR 'hospital units' OR 'hospitalization'/exp OR 'hospitalization' OR 'rehabilitation'/exp OR 'rehabilitation' OR 'hospitalised' OR 'hospitalized') NOT 'paediatric*' NOT 'pediatric*' NOT 'mental health unit' NOT 'intensive care unit' NOT 'covid-19' NOT 'coronavirus' NOT 'cancer care' NOT 'oncology' NOT 'palliative') AND ('sedentary lifestyle'/exp OR 'sedentary lifestyle' OR 'sedentary behaviour' OR 'sitting'/exp OR 'sitting' OR 'sedentary' OR 'physical activity'/exp OR 'physical activity' OR 'sedentary time'/exp OR 'sedentary time' OR 'sitting time' OR 'walked' OR 'walking'/exp OR 'walking' OR 'walks' OR 'exercising' OR 'exercise'/exp OR 'exercise' OR 'inactivity'/exp OR 'inactivity' OR 'mobility'/exp OR 'mobility') AND ('qualitative research'/exp OR 'qualitative research' OR 'qualitative'/exp OR 'qualitative' OR 'qualitatively' OR 'focus group'/exp OR 'focus group' OR 'interview'/exp OR 'interview' OR 'perspectives' OR 'perceptions' OR 'attitudes' OR 'perspective') AND [english]/lim AND [article]/lim |
| **PsycINFO/CINAHL/MEDLINE via EBSCOhost**  ( TI ( ("health personnel" OR "health care personnel" OR "health personnel" OR "health professionals" OR "health professional" OR "health care professional" OR "health care professionals" OR "healthcare professionals" OR "healthcare professional" OR "healthcare personnel" OR "allied health personnel" OR "allied health personnel" OR physiotherapist* OR "physical therapists" OR "physical therapist*" OR "occupational therapists" OR "occupational therapists" OR "occupational therapist" OR "physical therapy modalities" OR "physical therapy modalities" OR "physiotherapy" OR "physiotherapies" OR "physical therapy" OR "occupational therapy" OR "occupational therapy" OR "nurse s" OR "nurses" OR "nurses" OR "nurse" OR "nurses s" OR "Nursing" OR "physicians" OR "physicians" OR "doctor" OR "doctors" OR "exercise physiologist" OR "interdisciplinary studies" OR "multidisciplinary" OR multidiscipli* OR "clinician*") ) OR AB ( ("health personnel" OR "health care personnel" OR "health personnel" OR "health professionals" OR "health professional" OR "health care professional" OR "health care professionals" OR "healthcare professionals" OR "healthcare professional" OR "healthcare personnel" OR "allied health personnel" OR "allied health personnel" OR physiotherapist* OR "physical therapists" OR "physical therapist*" OR "occupational therapists" OR "occupational therapists" OR "occupational therapist" OR "physical therapy modalities" OR "physical therapy modalities" OR "physiotherapy" OR "physiotherapies" OR "physical therapy" OR "occupational therapy" OR "occupational therapy" OR "nurse s" OR "nurses" OR "nurses" OR "nurse" OR "nurses s" OR "Nursing" OR "physicians" OR "physicians" OR "doctor" OR "doctors" OR "exercise physiologist" OR "interdisciplinary studies" OR "multidisciplinary" OR multidiscipli* OR "clinician*") ) ) AND ( TI ( ( "acute care" OR "hospitalization" OR 'hospitalised' OR 'hospitalized' OR "acute care" OR "rehabilitation" OR "hospital") ) OR AB ( ( "acute care" OR "hospitalization" OR 'hospitalised' OR 'hospitalized' OR "acute care" OR "rehabilitation" OR "hospital") ) ) AND ( TI ( ( "sedentary behavior" OR "sedentary behaviour" OR "physical activity" OR "sitting time" OR "sitting" OR "walked" OR "walking" OR "walks" OR "exercising" OR "exercise" OR "inactivity" OR "mobility" OR “sedentary” ) ) OR AB ( ( "sedentary behavior" OR "sedentary behaviour" OR "physical activity" OR "sitting time" OR "sitting" OR "walked" OR "walking" OR "walks" OR "exercising" OR "exercise" OR "inactivity" OR "mobility" OR “sedentary” ) ) ) AND ( TI ( ( “Qualitative Research" OR "qualitative" OR "qualitatively" OR "focus group" OR "focus groups" OR "interview*" OR “attitudes” OR “perspective*” OR “perception*” ) ) OR AB ( ( “Qualitative Research" OR "qualitative" OR "qualitatively" OR "focus group" OR "focus groups" OR "interview*" OR “attitudes” OR “perspective*” OR “perception*” ) ) ) NOT ( TI ( ("paediatric*" OR "pediatric*" OR "mental health unit" OR "intensive care unit" OR "COVID-19" OR "coronavirus" OR "cancer care" OR "oncology" OR "palliative" ) ) OR AB ( ("paediatric*" OR "pediatric*" OR "mental health unit" OR "intensive care unit" OR "COVID-19" OR "coronavirus" OR "cancer care" OR "oncology" OR "palliative" ) ) ) |

| **Supplemental File 3:** Expanded study characteristics table | | | | | |
| --- | --- | --- | --- | --- | --- |
| **Authors** | **Country** | **Study aims** | **Methods** | **Setting** | **Participants** |
| Annemans, 2022^47^ | Belgium | To understand what defines, hampers, or supports physical activity in a rehabilitation centre | Focus groups | Free-standing inpatient rehabilitation centre | N=6  4 nurses & 2 therapists |
| Boltz, 2011^71^ | United States | To discover the organizational barriers and facilitators, as perceived by nursing staff, which influence physical function in this population. | Focus groups | Two hospitals: one suburban community hospital, one urban teaching hospital – various wards | N=55  55 nurses & PCAs (patient care associate)  Various areas of work: med-surg (n=36), emergency/critical care (n=7), specialty units (n=12) |
| Bor, 2022^48^ | Netherlands | To explore the perceived factors of influence on the implementation of interventions to improve patients’ movement behavior during hospitalization by health care professionals (HCPs) and patients. | Semi-structured interviews | 800 bed university teaching hospital: medical oncology*, haematology, cardiology & cardiothoracic surgery | N=16  9 nurses, 3 physical therapists & 4 unit managers  Medical oncology (n=4), haematology (n=4), cardiothoracic surgery (n=4) & cardiology (n=4) |
| Brown, 2007^49^ | United States | To identify barriers to mobility during hospitalization from the perspectives of older patients and their primary nurses and physicians, to compare and contrast the perceived barriers among these groups, and to make a conceptual model. | Semi-structured interviews | Hospital – medical ward | N=19  10 nurses & 9 physicians  All medical ward staff |
| Chan, 2019^72^ | Singapore | To explore nurses’ perceptions of older patients’ physical activity participation in an acute hospital setting in Singapore. | Focus groups | Tertiary hospital | N=30  Registered (n=25) and enrolled nurses (n=5) |
| De Klein, 2021^50^ | Netherlands | To explore the perspectives of both patients and health-care professionals on the amount of physical activity of hospitalized patients and what factors may promote the daily physical activity of patients. | Semi-structured interviews | University teaching hospital, geriatrics, and gastroenterology wards | N=9  Physiotherapists (n=2), nurses (n=4), doctors (n=3) |
| Doherty-King, 2011^73^ | United States | To explore how nurses make decisions about ambulating hospitalized older adults. | Semi-structured interviews | Two university teaching hospitals, adult medical and surgical wards | N=25  Registered nurses |
| Doherty-King, 2013^74^ | United States | To explore the relationship between nurses’ attributions of responsibility for ambulating hospitalized patients and their decisions about whether to ambulate. | Semi-structured interviews | Two university teaching hospitals, adult medical or surgical wards | N= 25  Registered nurses |
| Frederiksen, 2022^51^ | Denmark | To explore (1) the perspectives of health professionals on increased attendance of physical therapists (PTs) and occupational therapists (OTs) in clearly defined work assignments in two in-hospital departments and (2) the per- spectives of geriatric patients on physical activity during hospitalization. | Focus groups | Geriatric and infectious and pulmonary medical diseases departments in a medium-sized regional Danish hospital | N=8  Nurse assistants (n=2), nurses (n=2), physical therapists (n=2) & occupational therapists (n=2) |
| Geelen, 2021^52^ | Netherlands | To explore what healthcare professionals working at a university hospital consider to be the key barriers to improving physical activity in adults during hospital stay, and what solutions will help them to overcome these barriers | Semi-structured interviews and focus groups | University teaching hospital, five wards: gastrointestinal, oncology surgery, internal medicine haematology, infectious diseases and cardiology | N=30  Nurses (n=26), nursing assistant (n=1), physician assistant (n=1), physician (n=2) |
| Geidl, 2019^53^ | Germany | To explore the views of exercise therapists on physical activity promotion (PAP) and identify 1) the didactical–methodological approaches that exercise therapists use with the aim of promoting PA and 2) the facilitators and barriers that affect PAP. | Focus groups | Various facilities | N=58  Department heads with a background in physiotherapy (n=22), exercise therapy/sports science (n=38) or other (n=12), with n=47 working in inpatients and n=29 outpatients |
| Gustafson, 2021^54^ | United Kingdom | To explore the processes involved in post-ICU mobilisation through a human factors analysis, to inform future service improvement. | Focus group | Two hospitals | N=4  Ward physiotherapist (n=1), nurse researcher with ICU and ward experience (n=1), ICU physiotherapist (n=1), ICU follow up nurse (n=1) |
| Hazra, 2023^55^ | Canada | To measure the knowledge, attitudes, and practice patterns (KAP) of multidisciplinary cirrhosis providers about inhospital nutrition and physical activity care. | Online mixed methods survey | Thirty-eight hospitals and healthcare centres | N=338  Nurses (n=152), physicians (n=70), physiotherapists (n=12), occupational therapist (n=5), healthcare aide (n=8) & other professions (n=29) |
| Hills, 2021^75^ | Australia | To determine the factors influencing nurses’ decisions and capacity to reduce sedentary behaviour in hospital inpatients in sub-acute hospital settings. | Individual interviews | Inpatient subacute settings | N=11  Ward nurses (n=3), nurse leaders (n=3) |
| Janssen, 2022^56^ | Australia | To investigate rehabilitation staff perceptions of factors influencing stroke survivor activity outside of dedicated therapy time for the purpose of supporting successful translation of activity promoting interventions in a rehabilitation unit. | Semi-structured interviews | Four rehabilitation units (two mixed caseload, one neurological rehabilitation, one comprehensive stroke unit) | N=22  Nurses (n=4), nursing unit manager (n=1), rehabilitation physician (n=3), physiotherapist (n=5), occupational therapist (n=6), social worker (n=1), speech pathologist (n=3) |
| Jasper, 2023^57^ | Australia | To seek input from clinical staff from various health professions on strategies to increase physical activity and reduce sedentariness for hospitalised older people. | Focus group | Subacute geriatric ward and an acute orthopaedicward with an orthogeriatric service at a general hospital | N=7  Physiotherapists (n=2), occupational therapists (n=2), doctor (n=1), nurse (n=1) and social worker (n=1) |
| Johnson, 2019^58^ | United States | To explore the experience of patients, family members and staff on the unit during the mobility QI project in the acute care environment. | Semi-structured interviews | Community hospital, general medicine ward | N=8  Physicians (n=2), mobility technician (n=1), nurses (n=5) |
| King, 2016^76^ | United States | To determine whether all components of MOVIN can be implemented simultaneously and to test whether there was a change in nursing practice and unit culture after the intervention on one inpatient unit. | Focus groups and one semi-structured interview | University teaching hospital, general medicine ward | N=15  Registered nurses (n=10), certified nursing assistants (n=5) |
| Kirk, 2019^59^ | Denmark | To explore how social contextual circumstances affect the mobility of older medical patients in medical departments. | Go-along interviews during observation | Three hospitals: endocrinology, infectious diseases, and ED | N=79  Nursing assistants (n=16), registered nurses (n=33), physiotherapists (n=12), physicians (n=18) |
| Klooster, 2022^69^ | The Netherlands | To explain the perceived value of implementing a multifaceted intervention that aims to improve physically active behaviour in patients during hospital stays for healthcare professionals. | Semi-structured interviews | Various hospital wards | N=15  Physician (n=1), physiotherapists (n=2), nurses (n=10) and nurse assistants (n=2) |
| Kneafsey, 2015^77^ | United Kingdom | To explore the beliefs of the nursing team regarding hospital manual handling policy and the impact on the nursing contribution to promoting patients’ mobility | Semi-structured interviews | Three hospitals: general rehabilitation ward, spinal unit and stroke rehabilitation ward | N=33  Care support workers (n=11), registered nurses (n=15), ward sisters (n=7) |
| Kneafsey, 2013^60^ | United Kingdom | To present a grounded theory of the nursing team involvement in the process of maintaining and promoting the mobility of hospitalised older adults. | Semi-structured interviews | Three hospitals: general rehabilitation ward, spinal unit and stroke rehabilitation ward | N=39  Registered nurses (n=15), ward sisters (n=7), care support workers (n=10), therapy assistants (n=1), occupational therapist (n=2), physiotherapists (n=4) |
| Koenders, 2020^31^ | Netherlands | To understand beliefs, thoughts, attitudes, and experiences related to physical activity during hospital stay from the patients’ and health care providers’ perspective. | Semi-structured interviews | University hospital: cardiology, orthopaedics/trauma | N=24  Physical therapists (n=6), nurse assistants (n=6), nurses (n=8), physicians (n=3), physicians assistant (n=1) |
| Lim, 2020a^78^ | Singapore | To explore patients, their family carers, and nurses perceptions of promotion of mobility among hospitalised adults. | Semi-structured interviews | General medical ward of an acute care tertiary public hospital | N=10  Senior enrolled nurses (n=2), senior staff nurses (n=3), acting nurse clinician (n=1), nurse managers (n=4) |
| Lim, 2020b^61^ | United Kingdom | For the qualitative, staff component: to explore their [nurses and therapists] shared experience including barriers and facilitators of the [mobility] intervention. | Focus groups | Acute medical wards for older people | N=13  Therapists (n=7), nurses (n=6) |
| Lowe, 2018^79^ | United Kingdom | To develop understanding of physiotherapists' experience of PA promotion in UK physiotherapy practice | Semi-structured interviews | Varied amongst participants:  Primary care n=3, 25%  Secondary care n=3, 25%  Community n=2, 17%  A mixture n=4, 33% | N=12  Physiotherapists (n=12) |
| Moore, 2014^62^ | Canada | To assess clinicians’ knowledge about mobilization, the factors they perceived as facilitators and barriers to mobilization, and their capability and readiness to implement an early mobilization strategy | Focus groups | 26 hospital inpatient units | N=261  Eligible participants included nurses, nurse practitioners, occupational therapists, physiotherapists, physicians, managers, other allied health-care staff, and other unit staff such as personal support workers and ward clerks – however, professions of final participants not reported. |
| Myers, 2021^63^ | United Kingdom | To explore how the pilot was implemented, how it was experienced by Sports and Exercise Medicine (SEM) Consultants, healthcare professionals and patients, what worked well and what challenges were encountered | Semi-structured interviews | 2 clinical pathways included in the analysis: renal unit and complex medical unit | N=8  Sports and exercise medicine consultants (n=3), other healthcare professionals providing care in the renal (n=2) and complex medical unit (n=3) |
| Ohlsson-Nevo, 2020^80^ | Sweden | To describe how nurses perceive and promote inpatients' needs for physical activity during their stay at the ward | Focus groups | 7 medical & surgical wards in three hospitals, including one university hospital: surgical (4), medical (3), university hospital (3), smaller hospitals (4) | N=29  Registered nurses (n=15), certified nursing assistants (n=14) |
| Osinaike, 2021^81^ | United Kingdom | To explore the PA counselling attitudes and practices of junior doctors, to understand barriers to PA counselling practice among junior doctors and to understand enablers and facilitators to PA counselling among junior doctors | Semi-structured interviews | Various; participants worked across different settings, had spent at least 1 year in rotational foundation training. | N=11  Junior doctors in their second year of postgraduate medical practice (n=11) |
| Pavon, 2021^64^ | United States | To explore, using the socioecological model, the perspectives of both patients and hospital providers regarding: factors that influence hospital mobility in older adults, and, how these factors can inform the design or implementation of hospital walking or mobility programs | Semi-structured interviews with hospitalist physicians; focus groups with all other participants | General medicine ward at a tertiary medical centre and academic community hospital | N=48  Medical residents (n=7), nurses (n=9), certified nursing assistants (n=2), physical therapists (n=9), occupational therapists (n=11) |
| Pedersen, 2020^82^ | Denmark | To classify the most common barriers and facilitators to physicians' promotion of mobility in older medical patients as part of an intervention to promote mobility using the theoretical domains framework | Semi-structured interviews | Two medical departments of two hospitals | N=12  Physicians (n=12) |
| Pham, 2016^83^ | Vietnam | To describe nurses' conceptions about how health is promoted, with special focus on physically activity, for patients with type 2 diabetes and/or end-stage renal disease (ESRD). | Semi-structured interviews | Two major hospitals.  One specialised in geriatrics and patients with diabetes: study performed within the endocrinology department at this hospital  In the other hospital: study performed in the nephrology and urology department | N=25  Nurses (n=25) |
| Rasmussen, 2020^65^ | Denmark | To investigate the perspectives of health professionals on which factors may affect interventions, including physical exercise and nutrition, for frail older people in relation to discharge after acute admission to hospital. | Focus groups | Danish university hospital & a municipality | N=11  Physiotherapists (n=2), occupational therapists (n=2), social and healthcare assistant (n=4), home-care worker (n=1), nurses (n=2) |
| Scheerman, 2020^84^ | The Netherlands | To investigate how nurses perceive tasks and responsibilities in physical activity promotion of hospitalized older patients and which factors are of influence. | Semi-structured interviews | Academic teaching hospital, several wards: internal medicine; traumatology; oncological surgery; and a combined ward of vascular surgery, nephrology and urology. | N=51  Nurses (n=51, n=10 in supervisory roles) |
| Van Dijk-Huisman, 2022^70^ | The Netherlands | To explore and categorise patient- and health care professional-perceived barriers and enablers to physical activity behaviour in older adults admitted to a hospital with an acute medical illness, using the theoretical domains framework. | Semi-structured interviews | 715 bed combined university and regional hospital department of internal medicine | N=16  Nurses (n=6), physiotherapists (n=5) and physicians (n=5) |
| Williams, 2018^85^ | United Kingdom/Ireland | To explore the perceptions of physiotherapists in SCI rehabilitation on PA for people with SCI, and what is done to promote PA. | Semi-structured interviews | Regional spinal cord injury centres | N=18  Physiotherapists (n=18) |
| Wray, 2021^68^ | United Kingdom | To explore the experiences of older people and ward staff to identify modifiable factors (risk factors) which have the potential to reduce development or exacerbation of mechanisms of frailty during hospitalization (and physical and functional decline post-discharge). Using these experiences and informed by the literature, to then develop a theoretical framework of modifiable risk factors. | Focus groups | Two hospitals: one provides care for older adult inpatients only, and one combined orthogeriatric care and medical care for older adult inpatients | N=12  Doctors (n=3), staff nurses (n=1), trainee nurse (n=1), pharmacist (n=1), physiotherapist (n=1), occupational therapist (n=2), healthcare assistant (n=2) and technical instructor (n=1) |
| Wshah, 2021^66^ | Canada | To use the Theoretical Domains Framework (TDF) to explore the perspectives of HCPs regarding SB and strategies that could be considered to reduce it in people with COPD. | Semi-structured interviews | Pulmonary rehabilitation: inpatient and outpatients | N=16  Medical practitioner (n=2), physiotherapists (n=6), occupational therapists (n=3), nurses (n=3), respiratory therapists (n=2) |
| Zisberg, 2018^67^ | Israel | Overall aim of the paper: "to demonstrate the process of adapting a human factors framework, the Systems Engineering Initiative for Patient Safety (SEIPS 2.0), as a guided model to articulate a site-specific, culturally based intervention to improve in-hospital mobility in older adults."  For qualitative phase of interest: phase 1: "to explore organizational factors affecting in-hospital mobility.", phase 5: "to address barriers to mobility and to articulate the intervention protocol." | Interviews & focus groups | Two internal medicine units at an academic medical centre | Unclear total sample size, n=116 medical professions however multiphase approach, unclear sample size of qualitative component. |

**Supplemental File 4: Additional supporting quotes**

| **Central theme: Physical activity is not a priority** | |
| --- | --- |
| Subtheme 1: The hospital is a place for rest | “I have noticed that patients, when admitted to the hospital, even the patients who are mobile, immediately have the idea that they have to stay in bed all day in their pajamas” - Nurse^84^ |
|  | "There are also expectations of families, advocacy by protective families for reasons such as fear, grief, all of which have a role to play. What is considered reasonable? For example, ‘Dad’s in his 80s, does he need to do this?’ It is a common mindset of the family of an older person and there is generational impact." – Nurse leader^75^ |
|  | “I think nurses in general would prefer the patient to stay in bed. I believe they perceive it as a risk for falls and a risk for pulling out their IVs or any other medical device, and it [mobilising] is probably not viewed as an important factor in someone who is recovering from an illness.” - Nurse^49^ |
|  | "It is about keeping patients safe, but I do not think in rehab you can keep patients totally safe. There has to be risks, otherwise you don’t move them in bed all day because they are at risk of falling." - Nurse^77^ |
|  | "Some patients are really terribly ill. Those patients are for example extremely exhausted after sitting in a chair for thirty minutes. When somebody is ill like this, well ... They’re just unable to do anything." – Nurse^31^ |
|  | “For a patient if they are able to mobilise a little bit, instead of giving their food in the bedside, why can't you think about getting maybe a wall-mounted tray where it can fold out or lift it up and for lunch, they have to go up there to have their lunch...” – Social worker^57^ |
|  | “In general, I think older people always assume they have to stay in bed because they are sick” – Nurse^70^ |
| Subtheme 2: There are not enough resources to make movement a priority | "Reducing sedentary behaviour is an extra. So it’s not my priority." - Nurse^75^ |
|  | "If we had extra CNAs [certified nursing assistants], patients would get up faster and move more often, definitely." - Nurse^73^ |
|  | “Sometimes when patients return to the ward with a foreign equipment, like a drain, they don't dare to move or participate in ADL [activities of daily living]" - Nurse^72^ |
|  | " I notice recently patients are a lot more unwell, some patients taking 2, 3, 4 nurses to do basic nursing care. This takes all of those nurses away from other patients. It takes a lot of time to get them up, to take them for a walk." – Nurse^75^ |
|  | "...like if we’ve seen a patient and said they can walk ten metres, so they could walk to the bathroom, the health carers and the nurses are so busy on the ward that actually it’s easier to just transfer them onto a commode, wheel them to the bathroom because it saves time, so it’s not giving them the opportunity to mobilise, but because of staffing..." – Profession unknown^68^ |
|  | “When we have little time it’s just faster to take someone to the living room in a wheelchair. Usually this is caused by lack of time. However, many people would actually be able to walk there.” - Nurse^70^ |
| Subtheme 3: Everyone’s job is no one’s job | "I tell them they should get out of bed. But not like, ‘walk the stairs’ or something. I don’t know what to advise them. I leave that to the physiotherapist.” - Physician^50^ |
|  | "If I know that this 80-year-old woman played golf three times a week. . . you’re a little bit more aggressive with those people. Hey, you were out on the golf course before you came in with this emergency surgery so let’s get you back out on that golf course. " – Nurse^73^ |
|  | "I think we as physios, we are best placed to be the ones to educate and advise and encourage our patients to take part in physical activity ..." - Physiotherapist^85^ |
|  | “Yes, I think it’s important that the physician brings the message. I’ve experienced that if the nurse tells them to, not much happens, being they’re so authoritarian” - Physician^82^ |
|  | “Some don’t know what they’re allowed to and what they’re not allowed to” - Physician^82^ |
|  | "Going to a team meeting is good ... they say to the patient, this is what we are aiming for, do you agree that you will sit up for lunch every day ... it’s a team effort." - Nurse leader^75^ |
|  | “Everybody should be encouraging, everybody should be cheering the patients on, when they’re moving, when they’re improving. And reinforcing the benefits.” – Profession unknown^66^ |
|  | “There are really many reasons why it is important for patients to get out of bed, and I believe everyone here knows that and thinks it is an important thing here.” – Profession unknown^69^ |
|  | “And I’ve learned here that behavioural change... that you just have to take time for it. Because not everything that changes makes eve- rybody happy, and this takes a long time. You can move forward as an individual, but the rest also has to get there and that sometimes takes a few years.” – Profession unknown^69^ |
| Subtheme 4: Policy and leadership drives priorities | "Because of the hospital policies and protocols, we are very worried about fall risks should patients be allowed to participate in physical activity." - Nurse^72^ |
|  | "There are rules and procedures whereby patients should not pick up sheets by themselves in our closet. I have to pick it up for them" - Nurse^59^ |
|  | “When the unit manager and other administrators express that walking patients and having them stay physically and mentally active is important, it's easier to get the job done. Also, there is better teamwork when management supports this.” – Patient Care Associate^71^ |

**Supplemental File 5:** Quality appraisal summary using McMaster Qualitative Critical Review Form Version 2.0

| **Item** | | | | | | | | | | | | | | | | | | | | | | | | |  |
| --- | --- | --- | --- | --- | --- | --- | --- | --- | --- | --- | --- | --- | --- | --- | --- | --- | --- | --- | --- | --- | --- | --- | --- | --- | --- |
| **Study** | **1** | **2** | **3** | **4a** | **4b** | **4c** | **5a** | **5b** | **5c** | **5d** | **6** | **7a** | **7b** | **7c** | **7d** | **7e** | **8a** | **8b** | **8c** | **8d** | **9a** | **9b** | **Total** | **%*** | |
| Annemans 2022 | ● | ● | ○ | ○ | ◇ | ● | ● | ○ | ○ | ○ | ◇ | ● | ● | ◇ | ● | ● | ○ | ● | ○ | ○ | ● | ● | 11 | 58% | |
| Boltz 2011 | ● | ● | ○ | ● | ◇ | ● | ● | ● | ○ | ○ | ● | ● | ● | ● | ● | ● | ● | ● | ● | ● | ● | ● | 18 | 82% | |
| Bor 2022 | ● | ● | ● | ● | ● | ● | ● | ● | ● | ○ | ● | ● | ● | ◇ | ● | ● | ● | ● | ● | ● | ● | ● | 20 | 91% | |
| Brown 2007 | ● | ● | ● | ● | ● | ● | ● | ○ | ○ | ● | ● | ● | ● | ● | ● | ● | ○ | ○ | ● | ○ | ● | ● | 17 | 77% | |
| Chan 2019 | ● | ● | ○ | ● | ● | ● | ○ | ● | ○ | ○ | ● | ● | ● | ● | ● | ● | ● | ● | ● | ● | ● | ● | 18 | 82% | |
| DeKlein 2021 | ● | ● | ○ | ● | ● | ● | ● | ● | ○ | ○ | ○ | ● | ● | ◇ | ● | ● | ○ | ● | ○ | ○ | ● | ● | 14 | 64% | |
| Doherty-King 2011 | ● | ● | ○ | ● | ◇ | ◇ | ● | ○ | ○ | ○ | ○ | ● | ● | ◇ | ● | ● | ● | ○ | ● | ● | ● | ● | 13 | 59% | |
| Doherty-King 2013 | ● | ● | ● | ○ | ○ | ◇ | ● | ○ | ○ | ○ | ○ | ● | ● | ● | ● | ● | ● | ○ | ● | ● | ● | ● | 14 | 64% | |
| Frederiksen, 2022 | ● | ● | ○ | ● | ○ | ● | ● | ● | ● | ○ | ● | ● | ● | ◇ | ● | ● | ○ | ● | ● | ○ | ● | ● | 16 | 76% | |
| Geelen 2021 | ● | ● | ● | ● | ○ | ● | ● | ● | ● | ○ | ● | ● | ● | ● | ● | ● | ● | ● | ● | ● | ● | ● | 20 | 91% | |
| Geidl 2019 | ● | ● | ● | ● | ◇ | ◇ | ○ | ● | ● | ○ | ● | ● | ● | ● | ● | ● | ○ | ○ | ○ | ● | ● | ● | 15 | 68% | |
| Gustafson 2021 | ● | ● | ○ | ● | ◇ | ◇ | ● | ○ | ○ | ○ | ● | ○ | ○ | ◇ | ○ | ● | ○ | ○ | ○ | ○ | ● | ● | 8 | 36% | |
| Hazra, 2023 | ● | ● | ○ | ● | ◇ | ● | ● | ● | ○ | ○ | ● | ● | ● | ◇ | ● | ● | ○ | ○ | ○ | ○ | ● | ● | 13 | 65% | |
| Hills 2021 | ● | ● | ○ | ● | ◇ | ● | ● | ○ | ○ | ○ | ○ | ● | ● | ◇ | ● | ● | ○ | ○ | ● | ○ | ● | ● | 12 | 55% | |
| Janssen 2022 | ● | ● | ○ | ● | ● | ◇ | ● | ● | ● | ○ | ● | ● | ● | ● | ● | ● | ● | ● | ● | ● | ● | ● | 19 | 86% | |
| Jasper, 2023 | ● | ● | ● | ● | ◇ | ● | ● | ○ | ○ | ○ | ● | ● | ● | ◇ | ● | ● | ○ | ● | ○ | ○ | ● | ● | 14 | 70% | |
| Johnson 2019 | ● | ○ | ○ | ● | ◇ | ● | ● | ○ | ● | ● | ○ | ● | ● | ● | ● | ● | ● | ○ | ● | ● | ● | ○ | 15 | 68% | |
| King 2016 | ● | ● | ● | ● | ◇ | ◇ | ● | ○ | ○ | ○ | ● | ● | ● | ◇ | ● | ● | ○ | ○ | ○ | ○ | ● | ● | 12 | 55% | |
| Kirk 2019 | ● | ● | ● | ● | ◇ | ○ | ● | ○ | ○ | ● | ● | ● | ● | ◇ | ● | ● | ● | ● | ● | ● | ● | ● | 17 | 77% | |
| Klooster, 2022 | ● | ● | ○ | ● | ● | ● | ● | ● | ● | ○ | ● | ● | ● | ◇ | ● | ● | ● | ● | ● | ● | ● | ● | 19 | 90% | |
| Kneafsey 2013 | ● | ● | ○ | ● | ● | ● | ● | ● | ○ | ○ | ● | ● | ● | ● | ● | ● | ● | ● | ● | ○ | ● | ● | 18 | 82% | |
| Kneafsey 2015 | ● | ● | ● | ● | ◇ | ● | ● | ○ | ○ | ○ | ● | ● | ● | ◇ | ● | ● | ○ | ○ | ○ | ○ | ● | ● | 13 | 59% | |
| Koenders 2020 | ● | ● | ○ | ● | ● | ● | ● | ● | ● | ○ | ● | ● | ● | ● | ● | ● | ● | ● | ● | ● | ● | ● | 20 | 91% | |
| Lim 2020 | ● | ● | ○ | ● | ● | ● | ● | ● | ○ | ○ | ● | ● | ● | ● | ● | ● | ● | ● | ● | ● | ● | ● | 19 | 86% | |
| Lim 2020 | ● | ● | ● | ● | ◇ | ◇ | ● | ○ | ○ | ○ | ○ | ◇ | ● | ◇ | ○ | ● | ○ | ○ | ○ | ○ | ● | ● | 9 | 41% | |
| Lowe 2018 | ● | ● | ● | ● | ● | ● | ○ | ○ | ● | ● | ● | ● | ● | ● | ● | ● | ● | ○ | ● | ● | ● | ● | 19 | 86% | |
| Moore 2014 | ● | ● | ● | ● | ◇ | ◇ | ○ | ○ | ○ | ○ | ● | ● | ● | ◇ | ● | ● | ● | ○ | ○ | ○ | ● | ● | 12 | 55% | |
| Myers 2021 | ● | ● | ○ | ● | ◇ | ● | ● | ○ | ● | ○ | ● | ● | ● | ◇ | ● | ● | ● | ○ | ● | ● | ● | ● | 16 | 73% | |
| Ohlsson-Nevo 2020 | ● | ● | ● | ● | ◇ | ● | ● | ● | ● | ● | ● | ● | ● | ● | ● | ● | ● | ● | ● | ● | ● | ● | 21 | 95% | |
| Osinaike 2021 | ● | ● | ○ | ● | ● | ● | ● | ● | ○ | ○ | ● | ● | ● | ◇ | ● | ● | ○ | ● | ● | ○ | ● | ● | 16 | 73% | |
| Pavon 2021 | ● | ● | ● | ● | ● | ◇ | ● | ● | ○ | ○ | ● | ○ | ● | ● | ● | ● | ● | ● | ● | ● | ● | ● | 18 | 82% | |
| Pedersen 2020 | ● | ● | ● | ● | ○ | ● | ● | ● | ● | ○ | ● | ○ | ● | ◇ | ● | ● | ● | ● | ● | ● | ● | ● | 18 | 82% | |
| Pham 2016 | ● | ● | ○ | ● | ◇ | ● | ● | ● | ● | ○ | ● | ● | ● | ◇ | ● | ● | ● | ● | ● | ○ | ● | ● | 17 | 77% | |
| Rasmussen 2020 | ● | ● | ○ | ● | ◇ | ● | ● | ● | ● | ○ | ● | ● | ● | ◇ | ● | ● | ● | ● | ○ | ● | ● | ● | 17 | 77% | |
| Scheerman 2020 | ● | ● | ○ | ● | ◇ | ● | ● | ● | ● | ○ | ● | ○ | ● | ◇ | ● | ● | ○ | ● | ● | ○ | ● | ● | 15 | 68% | |
| Van Dijk-Huisman, 2022 | ● | ● | ○ | ● | ● | ● | ● | ● | ● | ○ | ● | ○ | ● | ● | ● | ● | ● | ● | ● | ● | ● | ● | 19 | 86% | |
| Williams 2018 | ● | ● | ● | ● | ● | ◇ | ○ | ● | ○ | ○ | ● | ● | ● | ● | ● | ● | ● | ● | ● | ● | ● | ● | 18 | 82% | |
| Wray 2021 | ● | ● | ● | ● | ● | ● | ● | ○ | ● | ○ | ● | ○ | ● | ● | ● | ● | ● | ○ | ● | ● | ● | ● | 18 | 82% | |
| Wshah 2021 | ● | ● | ● | ● | ● | ● | ● | ● | ● | ○ | ● | ○ | ● | ◇ | ● | ● | ● | ● | ● | ● | ● | ● | 19 | 86% | |
| Zisberg 2018 | ● | ● | ● | ● | ◇ | ● | ● | ○ | ○ | ○ | ○ | ○ | ● | ◇ | ○ | ● | ○ | ○ | ○ | ○ | ● | ● | 10 | 45% | |

Notes: Unfilled circle (**○**) indicates no point, filled circle indicates point awarded (●), diamond indicates not addressed (◇)
Items scored as follows (points possible):
1. Study purpose (1)
2. Literature review (1)
3. Theoretical perspective identified (1)
4. Sampling: a. Process of purposeful selection described? (1) b. Sampling done until redundancy in data? (1) c. Informed consent obtained? (1)
5. Data collection: a. Clear and complete description of site (1) and b. of participants (1) c. Role of researcher & participants (1) d. Identification of assumptions & biases of researcher (1)

6. Procedural rigour (1)
7. Data analyses: a. Data analyses were inductive (1) b. Findings were consistent with & reflective of data? (1) c. Decision trail developed? (1) d. Process of data analysis described adequately? (1) e. Meaningful picture of phenomenon emerged? (1)
8. Overall rigour: a. Credibility (1) b. Transferability (1) c. Dependability (1) d. Confirmability (1)
9. Complications & implications: a. Conclusions appropriate given findings? (1) b. Findings contributed to theory development and future practice/research? (1)
* Percentage calculated as points awarded/points possible, without inclusion of items marked as “not addressed”, presented as a whole number
